# Biomethane Production from Stillage: The Role of Temperature, Initial Substrate Load, and Inoculum Variability

**DOI:** 10.1021/acsomega.5c03798

**Published:** 2025-09-18

**Authors:** Tomáš Vítěz, Nikola Hanišáková, Pavel Suchý, Petra Tejchmanová, Jan Kudělka, Tomáš Koutný, Jan Lochman, David Novák, Monika Vítězová

**Affiliations:** † Department of Agricultural, Food and Environmental Engineering, Faculty of AgriSciences, 48269 Mendel University in Brno, Zemedelska 1, 613 00 Brno, Czech Republic; ‡ Department of Experimental Biology, Section of Microbiology, Faculty of Science, Masaryk University, Kamenice 753/5, 625 00 Brno, Czech Republic; § Department of Biochemistry, Faculty of Science, Masaryk University, Kamenice 753/5, 625 00 Brno, Czech Republic

## Abstract

Molasses stillage, with its high organic content and nutrient-rich composition, represents a promising feedstock for biogas production. This study systematically evaluated its biochemical methane potential (BMP) using two inocula (wastewater treatment plant vs agricultural biogas plant) across three temperatures (40, 50, 60 °C) and initial substrate load (ISL: 2, 5, 10 g·L_incolum_^–1^). The wastewater inoculum achieved superior methane yields (0.262–0.477 N m^3^·kg_vs_^–1^), peaking at 50 °C with a 22% increase over agricultural systems (0.192–0.378 N m^3^·kg_vs_^–1^). 16S rRNA sequencing revealed the wastewater treatment plant inoculum’s superior functional diversity, dominated by syntrophic *Chloroflexota* and *Acidobacteriota* alongside methanogenic *Methanobacterium* (52–61% relative abundance) and acetoclastic *Methanothrix* (18–23%). In contrast, agricultural biogas plant inocula showed specialized thermophilic communities dominated by *Bacillota* (68–72%) and hydrogenotrophic *Methanoculleus* (29–34%). Both systems exhibited inhibition at 60 °C/ISL10 (yields reduced by 34–42%), correlating with declining diversity and *Methanofastidiosum* proliferation. These findings provide two key operational insights: wastewater inocula offer greater process stability due to microbial diversity, and 50 °C represents the thermal optimum for stillage codigestion, balancing yield and community resilience.

## Graphical abstract

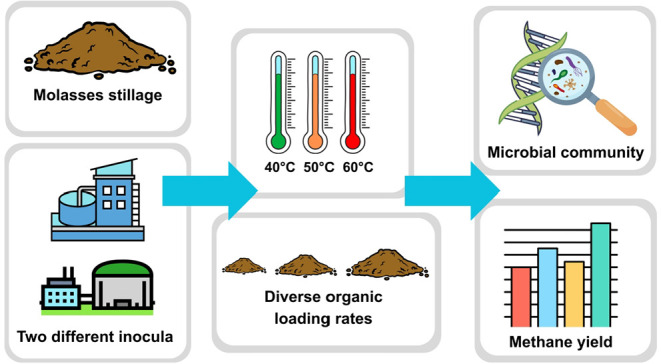

## 1 Introduction

Distillery stillage is an unavoidable byproduct of ethanol production generated during fermentation and subsequent distillation. Due to its high content of suspended solids and inhibitory compounds (e.g., glycerol, lactic acid, and acetic acid), which are toxic to yeast, stillage is typically not recycled in ethanol production and instead becomes a waste stream.^1^ Approximately 20 L of stillage is produced per liter of ethanol, depending on feedstock and processing conditions. Molasses stillage is a dark brown, acidic (pH 3.5–5.0) effluent composed of water, organic/inorganic compounds, and residual fermentation products. Its properties—including high biological oxygen demand (BOD: 40–70 g·L^–1^), chemical oxygen demand (COD: 80–122 g·L^–1^), and volatile solids (up to 60 g·L^–1^)—are influenced by feedstock and production technology.^2,3^ Notably, stillage from sugar cane or sugar beet molasses may contain 10-fold higher sulfate concentrations than other feedstocks, attributed to sulfuric acid use in pH adjustment or sugar clarification.^4^ The dry matter of molasses stillage (4.1–12.4%) consists largely of organic material (66–91%), including proteins, melanoidins, and organic acids. However, it may also contain hazardous contaminants, such as phenols, polyphenols, and heavy metals. Among heavy metals, concentrations of copper (2.2–37.8 mg·kg^–1^), lead (0.5–8.8 mg·kg^–1^), zinc (2.7–47.7 mg·kg^–1^), and some other metals such as arsenic, cadmium, and mercury can be detected.^5,6^

Nowadays, there are many uses for molasses stillage, where this matrix can be used for the production of other chemicals such as chitosan, astaxanthin, calcium magnesium acetate, and some enzymes and biopolymers (alternan, pullulan), but also for the production of biochar, agricultural fertilizers, and livestock feed.^7–9^ Energy recovery is also a promising approach, especially as a feedstock for biogas plants. Anaerobic treatment of distillery stillage for biogas production has been discussed in studies since the 1980s. Although distillery stillage does not seem to be an ideal material for anaerobic digestion due to its composition, it is possible to produce 20.7–22.5 m^3^ of biogas from 1 m^3^ of stillage, with methane concentration in the produced biogas in the range of 50–70 vol %.^10^ Process temperature during anaerobic fermentation has a significant impact on biogas production and has been intensively studied.^11,12^ However, the anaerobic processing of distillery stillage alone brings several problems related to its properties. For example, molasses stillage has a low C/N ratio (<15), low buffering capacity, and relatively low content of trace elements (Co, Ni, Fe) necessary for the development of anaerobic organisms, so these elements must be supplied artificially.^6^ Another problem can be the higher sulfate concentration in this material, which can lead to the growth of sulfate-reducing bacteria (SRB) during fermentation, which use the same substrate as the methanogenic archaea in their sulfate metabolism, resulting in a decrease in methane production.^13^ If SRB becomes prevalent, H_2_S can subsequently accumulate in the system, which will greatly impact the life of the materials used in the technology due to its corrosive effect. Another problematic component of stillage is the high concentration of proteins that, when degraded, release nitrogen in the form of ammonium (NH_4_^+^), which is toxic to methanogenic archaea. In conjunction with protein degradation, increasing concentrations of sulfides are also expected due to the degradation of the amino acids cysteine and methionine. Due to these facts, it is more suitable to use molasses stillage for cofermentation in anaerobic reactors either in wastewater treatment plants or in agricultural biogas plants, where the ideal materials for cofermentation are sewage sludge or cow dung containing the necessary trace elements (Co, Ni) and buffer components. Understanding microbial dynamics and key metabolic pathways is essential for optimizing methane production during cofermentation, as both microbial interactions and enzyme activity critically influence biogas yields.^14^ According to some authors, 5–15% of the world energy consumption in the distillery industry could be covered by this method of processing distillery stillage, regardless of the raw materials used and the processing technology. Moreover, this method of processing distillery stillage fits into the concept of circular economy, in which more emphasis is placed on sustainability and climate, which puts pressure on modern methods of processing this raw material.

While anaerobic digestion offers attractive valorization potential, critical knowledge gaps remain regarding optimal process parameters and inoculum selection. Specifically, previous studies have not comprehensively compared wastewater vs agricultural inocula across relevant temperature ranges (40–60 °C) while accounting for initial substrate load (ISL) effects on microbial community function and process stability. Our study addresses these gaps through systematic evaluation of two distinct inocula (wastewater Modrice vs agricultural Cejc) across multiple ISLs (2–10 g_VS_/L), combining advanced process monitoring with functional microbial analysis (FAPROTAX). The results demonstrate the wastewater inoculum’s superior performance at 50 °C (15–22% higher yields than agricultural systems) while revealing shared inhibition thresholds at 60 °C/ISL10, providing practical insights for optimizing stillage codigestion in biogas plants.

## 2 Materials and Methods

### 2.1 Analytical Methods

The total solids content was determined by drying the samples in an electric laboratory dryer, KBS G 100 (Premed, Poland), at 105 ± 3.5 °C to constant weight according to SN EN 15934. Volatile solids content was determined by incineration of a sample in an LMH 07/12 muffle furnace (LAC, Idlochovice, Czech Republic) at 550 ± 10 °C to constant weight according to SN EN 15935. The pH, oxidation–reduction potential, and conductivity were measured with a Greisinger GHM 5530 handheld pH meter using the GE 100, GR 105, and LF 400 electrodes (GHM Messtechnik GmbH, Remscheid, Germany). The sample for calorific value determination was weighed on a Pioneer PA4102C laboratory balance (OHAUS CORPORATION, Parsippany, NJ). The calorific value was determined by using a Parr 6400 calorimeter (Parr Instrument Company, Moline, IL). Screening tests of the elemental content of the molasses stillage were performed by using a Niton XL3t X-ray spectrometer (Thermo Fisher Scientific, Waltham, MA).

### 2.2 Biochemical Methane Potential Tests

The biochemical methane potential (BMP) tests were conducted according to the modified standard VDI 4630:2016. Three systems with a total of 24 batch digesters were used for the tests. A simplified schematic of the laboratory custom-made system for the BMP tests is shown in Figure 1.

**Figure 1:**
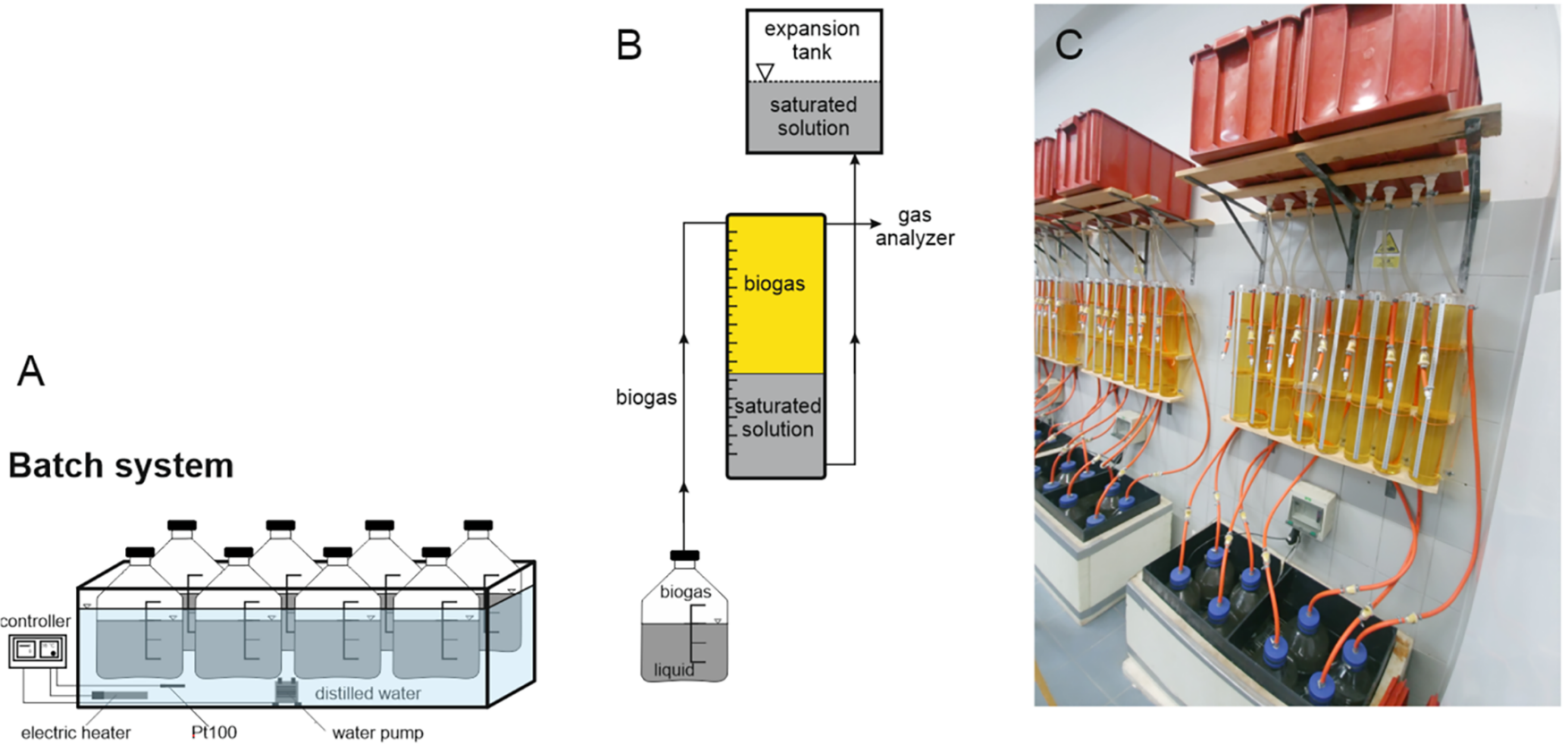
Schematic of the laboratory system for the BMP tests; (A) water bath with eight fermenters and a controller; (B) detailed view of the fermenter connection; (C) overall view of the system with eight fermenters.

A set of eight 5 L glass fermenters was placed in three separate water baths. The temperature of the water in the water baths was maintained at the desired temperature by an electric heater and controlled by a Resistance Temperature Detector Pt100. On the first day of the experiment, 3 kg of sieved inoculum (3 mm, mesh size) was added to each fermenter. Then, a sample of the molasses stillage was added to 6 fermenters (the dosage is shown in Table 1). The remaining two fermenters were used as blanks in which the endogenous methane production of the inoculum was determined. Each fermenter was connected to a glass measuring cylinder via a hose. The biogas produced forced the salt-saturated solution from the glass cylinder into an expansion vessel. Every day, the biogas production was subtracted from the scale of the measuring cylinder. The biogas accumulated in the glass cylinder could be analyzed using the quick-coupling sampling point integrated into each cylinder. The gas analyzer X-am 8000 (Dräger, Lübeck, Germany) was used to analyze the composition of the biogas produced. Methane production was converted to normal conditions (*p* = 1 bar; *T* = 273.15 K). The endogenous methane production of the inoculum was subtracted from the final methane production of each fermenter. All tests were carried out until the daily biogas yield in three consecutive days was <1% of the total biogas yield (VDI 4630). Methane production is expressed in m^3^ CH_4_ per kg of volatile solids added.

**Table 1:** Characteristics of Inocula and BMP Tests

	parameter	value
inoculum 1	the place of collection	WWTP Modrice, Czech Republic; anaerobic sewage sludge stabilization; mesophilic temperature conditions (38 °C); substrate: mixture of primary and waste-activated sludge (50/50, w/w %)
	total solids [%]	3.42 ± 0.04
	volatile solids [%_TS_]	56.69 ± 0.08
	pH	7.1 ± 0.10
	other parameters FOS [mg·L^–1^]	FOS 780 mg·L^–1^; TAC 2400 mg·L^–1^; NH_4_^+^ 560 mg·L^–1^
inoculum 2	the place of collection	agriculture biogas plant; Cejc, Czech Republic; mesophilic temperature conditions (40 °C); substrate: mixture of maize silage and pig manure (80/20, w/w %)
	total solids [%]	3.98 ± 0.08
	volatile solids [%_TS_]	73.79 ± 0.10
	pH	6.9 ± 0.09
	other parameters FOS [mg·L^–1^]	FOS 690 mg·L^–1^; TAC 2000 mg·L^–1^; NH_4_^+^ 460 mg·L^–1^
biomethane potential test	fermenter volume	total volume 5 dm^3^; working volume 3 dm^3^
	temperature [°C]; heating method	40 °C; 50 °C; 60 °C ± 0.2 °C; water Baths
	agitation	manually, daily
	residence time	All tests were carried out until the daily biogas yield in 3 consecutive days was <1% of the total biogas yield, 21 days
	stillage dose [g]	14; 40; 80
	initial substrate load (ISL) [g_VS_·L_inoculum_^–1^]	2; 5; 10
	method of measuring biogas production	Liquid expansion method, according to VDI 4630
	method of measuring biogas composition	Dräger X-am 8000 gas analyzer: infrared sensors for CH_4_ and CO_2_, calibration gas mixture (60% CH_4_/40% CO_2_)
	number of repetitions	2 for each substrate load

Two inoculum sources were used to test the BMP. The first inoculum was sewage sludge from anaerobic sewage sludge stabilization of the wastewater treatment plant (WWTP) in Modrice, Czech Republic. The second inoculum was the digestate from the agricultural biogas plant Cejc, Czech Republic. The characteristics of the inocula and substrate used in the tests are shown in Table 1.

The theoretical methane yield was calculated using elemental analysis data and Buswell’s stoichiometric formula as described in ref 15. The biodegradability index (BI) was calculated as the ratio of experimental BMP to theoretical yield using the formula described in refs 16,17 .

### 2.3 Tested Substrate

Molasses stillage from the distillery Tereos TTD, a.s., Lihovar Kojetin (Czech Republic) served as the substrate for all BMP tests. Its composition is detailed in Table 2.

**Table 2:** Composition of the Molasses Stillage^a^

parameter	value	parameter	value [ppm]
total solids [%]	47.6	Mg [g·kg^–1^]	0.6
volatile solids [%_TS_]	73.2	Na [g·kg^–1^]	30.7
pH	6.1	S [g·kg^–1^]	8.8
TOC [g·kg^–1^]	0.0399	As [mg·kg^–1^]	0.292
C:N	6.4	Cd [mg·kg^–1^]	<0.034
density [kg·m^–3^]	1232	Cr [mg·kg^–1^]	0.312
*N*_tot_ [%]	5.71	Cu [mg·kg^–1^]	1.28
P [g·kg^–1^]	0.5	Hg [mg·kg^–1^]	0.013
K [g·kg^–1^]	87.3	Mo [mg·kg^–1^]	0.389
S [g·kg^–1^]	8.8	Ni [mg·kg^–1^]	5.47
SO_4_^2–^ [g·kg^–1^]	12.6	Pb [mg·kg^–1^]	<0.293
Cl^–^ [g·kg^–1^]	18.5	Zn [mg·kg^–1^]	63.7

a Results of the analysis of molasses distillation residues from an accredited laboratory.

To prevent degradation that could influence BMP test results, the stillage was stored at 4 °C between test intervals. Table 3 presents the effects of long-term storage on its properties, along with indicative compositional measurements of the stillage used in the BMP assays.

**Table 3:** Effect of Long-Term Storage on the Properties of the Molasses Stillage^a^

parameter	sample of stillage	sample of stillage (after 11 months)	sample of stillage (after 23 months)
total solids [%]	49.89 ± 1.02	47.64 ± 0.25	48.63 ± 0.34
volatile solids [%_TS_]	73.61 ± 0.08	72.51 ± 1.02	73.03 ± 0.20
pH	5.84 ± 0.07	6.02 ± 0.01	5.99 ± 0.02
conductivity [μS·cm^–1^]	29,926.7 ± 752.95	31,223.3 ± 63.42	29,823.3 ± 20.55
gross calorific value [MJ·kg^–1^]	16.12 ± 0.42	15.33 ± 0.33	ND

a Mean of measured values (*n* = 3) is shown; ±standard deviation (SD); ND—not determined.

### 2.4 Molecular Biological Methods

#### 2.4.1 Sample Processing

After confirming stable methane production across all temperatures by day 21 of cultivation, 50 mL samples were collected from each fermenter in conical centrifuge tubes. DNA was extracted using the QIAamp PowerFecal Kit (Qiagen, Germany) following manufacturer protocols, with quality verified using a NanoDrop 2000 spectrophotometer (Thermo Fisher Scientific). All DNA extracts were stored at −20 °C for subsequent analysis.

After DNA isolation, amplification of the hypervariable region V4 of the 16S rRNA gene was performed. For this reaction, the following primers were used enabling the dual-index bar-coding method: 515F Forward primer 5′-AATGATACGGCGACCACCGAGATCTACACTATGGTAATTGTGTGCCAGCMGCCGCGGTAA-3′ and 806R Reverse primer 5′-CAAGCAGAAGACGGCATACGAGATAGTCAGTCAGCCGGACTACHVGGGTWTCTAAT-3′.^18^ PCR amplification was performed according to the Earth Microbiome Project protocol^19^ in a reaction volume of 25 μL with 10 μL Platinum II Hot-Start PCR Master Mix (Thermo Fisher Scientific), 200 nM primers, and 2 μL isolated DNA. The PCR was performed as described previously.^20^ PCR products were purified with AMPure XP Beads (Beckman Coulter, Brea, CA), quantified using Qubit 4 (Thermo Fisher Scientific, Waltham, MA), and quality-checked with a Fragment Analyzer (Agilent Technologies, Santa Clara, CA). The final library was sequenced on an Illumina MiniSeq (Illumina, San Diego, CA) using MidOutput Reagent Kits (2 × 150 bp).

#### 2.4.2 Sequences Analyses

The raw sequencing data were processed using the DADA2 package (v1.16),^21^ which included quality filtering (removing ambiguous bases, truncating reverse reads by 1 bp, and applying an expected error threshold of 2), dereplication, denoising, read merging, and chimera removal.^22,23^ Taxonomy was assigned using the RDP classifier against the SILVA NR v138.2 database,^24^ resulting in a high-resolution amplicon sequence variant (ASV) table. For subsequent analyses, all samples were rarefied to 38900 reads. The final processed dataraw fastq files are available under BioProject PRJNA1218928.

### 2.5 Data Processing

Using experimental data, the basic statistical parameters (M-mean, SD-standard deviation, and M ± SD) were calculated. The plots were built by software package OriginPro 2023 (OriginLab Corporation, Northampton, MA) and using R (version 4.2.3) within RStudio environment (2024.09.0 + 375). The taxonomic data were analyzed by microeco package (version 1.2.2).^25^ Functional prediction of metabolic pathways was processed through microeco package function trans_func, using FAPROTAX database (version 1.2.10).^26^

## 3 Results and Discussion

Figure 2 presents methane yields across different initial substrate loads (ISL) and temperatures for both inocula. The 50 °C condition consistently demonstrated optimal performance, with the agricultural biogas inoculum (Cejc) achieving peak yields of 0.367–0.378 N m^3^·kg_vs_^–1^ at ISL2 and ISL5 (Figure 2A), while the wastewater treatment inoculum (Modrice) reached higher maxima (0.414–0.447 N m^3^·kg_vs_^–1^) at ISL2 and ISL10 (Figure 2B). Both systems exhibited declining yields with increasing ISL at 50 °C and pronounced inhibition under combined high-temperature/high-load conditions (60 °C with an ISL ≥ 5), confirming that excessive substrate loading exacerbates thermal stress. Statistical analyses revealed distinct thermal optima for each inoculum (*t* tests, α=0.05). The wastewater treatment inoculum (Modrice) achieved significantly higher methane yields at 50 °C compared to both 40 °C (*p* = 0.028) and 60 °C (*p* = 0.010), with peak production (0.414–0.447 N m^3^·kg_vs_^–1^) at ISL10 and ISL2 (Figure 2B). In contrast, the agricultural inoculum (Cejc) showed only a single significant temperature effect (yield reduction at 60 °C vs 50 °C (*p* = 0.014)), with no differences between 40 °C/50 °C (*p* = 0.100) or 40 °C/60 °C (*p* = 0.067). Both inocula exhibited declining yields with increasing ISL at 50 °C and pronounced inhibition under combined high-temperature/high-load conditions (60 °C with an ISL ≥ 5). However, Modrice maintained consistently superior performance, with 15–22% higher absolute yields (0.262–0.447 N m^3^·kg_vs_^–1^ vs 0.192–0.378 N m^3^·kg_vs_^–1^ after 21 days) and faster kinetics (stationary phase reached by day 4 vs day 7 for Cejc). These results demonstrate that while 50 °C represents a universal optimum, the wastewater inoculum’s adaptation to complex substrates confers enhanced process efficiency and robustness.

**Figure 2:**
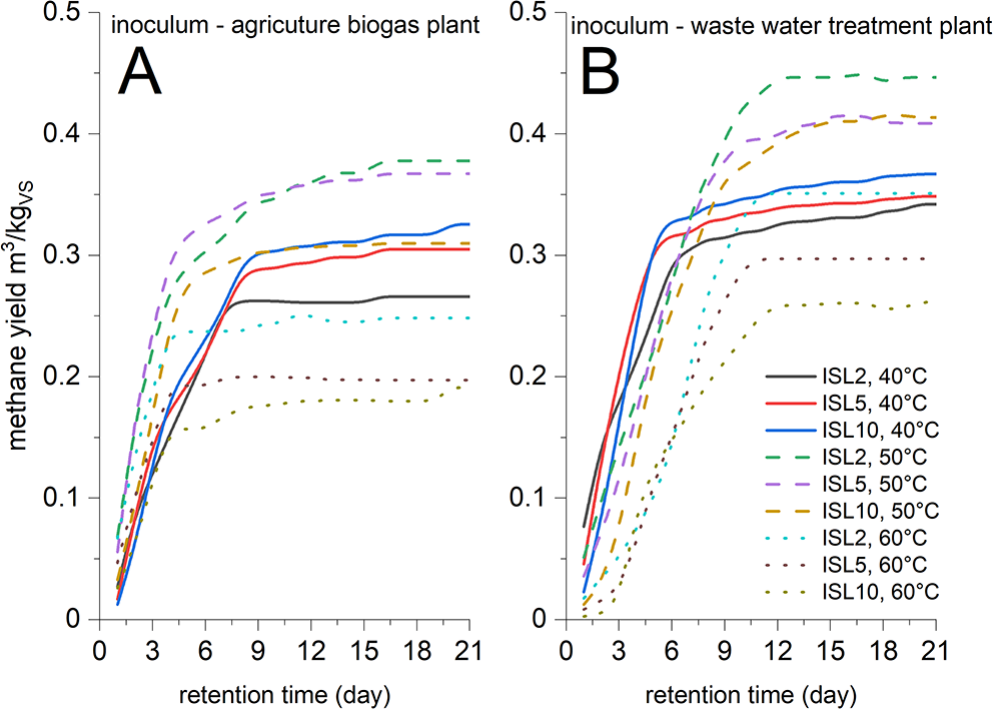
Methane yield during biomethane potential tests; (A) inoculum Cejc (agricultural biogas plant); (B) inoculum Modrice (wastewater treatment plant).

The determined methane yields during fermentation of molasses stillage are in accordance with values published by other authors: Ziemiński and Kowalska-Wentel^27^ 0.277 N m^3^·kg_vs_^–1^; Moraes et al.^28^ 0.267 N m^3^·kg_vs_^–1^; Wang et al.^29^ 0.311 N m^3^·kg_vs_^–1^; Dubrovskis and Plume^30^ 0.360 N m^3^·kg_vs_^–1^; and Zubrowska-Sudol et al.^31^ 0.312 N m^3^·kg_vs_^–1^. Biogas quality was also monitored during fermentation regarding H_2_ and H_2_S concentrations. No increasing H_2_ concentrations were observed during the BMP tests, which would be a clear signal of a biological problem in the system, especially the inhibition of hydrogen-metabolizing microorganisms. During the BMP tests, there was also no accumulation of H_2_S, which commonly occurs during the fermentation of materials that are rich in protein, such as molasses stillage. H_2_S concentrations during 21 days of fermentation ranged from 4 to 126 ppm in samples with inoculum Cejc (agricultural biogas plant), whereas zero concentrations of H_2_S were detected in samples with inoculum Modrice (wastewater treatment plant).

The biodegradability index (BI) was calculated as the ratio of experimental to theoretical methane yield (0.51 N m^3^/kg_VS_, derived via Buswell’s equation). For the wastewater inoculum (Modrice), optimal performance occurred at 50 °C across all the ILSs (BI = 0.80–0.88), while 60 °C showed marked ILS dependence (BI = 0.51–0.69). In contrast, the agricultural inoculum (Cejc) exhibited lower overall BI values (0.38–0.74), with the strongest performance at 50 °C/ILS2 (BI = 0.72) and notable inhibition at 60 °C/ILS5–10 (BI = 0.38–0.39). Both inocula maintained stable mesophilic functionality (40 °C BI = 0.60–0.72), but Modrice demonstrated superior thermophilic adaptability (60 °C BI + 31% vs Cejc) and ILS resilience, particularly at higher loads. These differences likely reflect the wastewater consortium’s enhanced stress tolerance and metabolic flexibility.

Figure 3 illustrates the variation in the methane content of biogas produced during the BMP tests. The biogas generated from samples inoculated with material from the wastewater treatment plant exhibited higher methane concentrations, ranging from 71 to 71.5 vol % after 21 days of BMP. Methane concentrations in biogas from samples inoculated with material from the agricultural biogas plant ranging from 61 to 65 vol % after 21 days of BMP.

**Figure 3:**
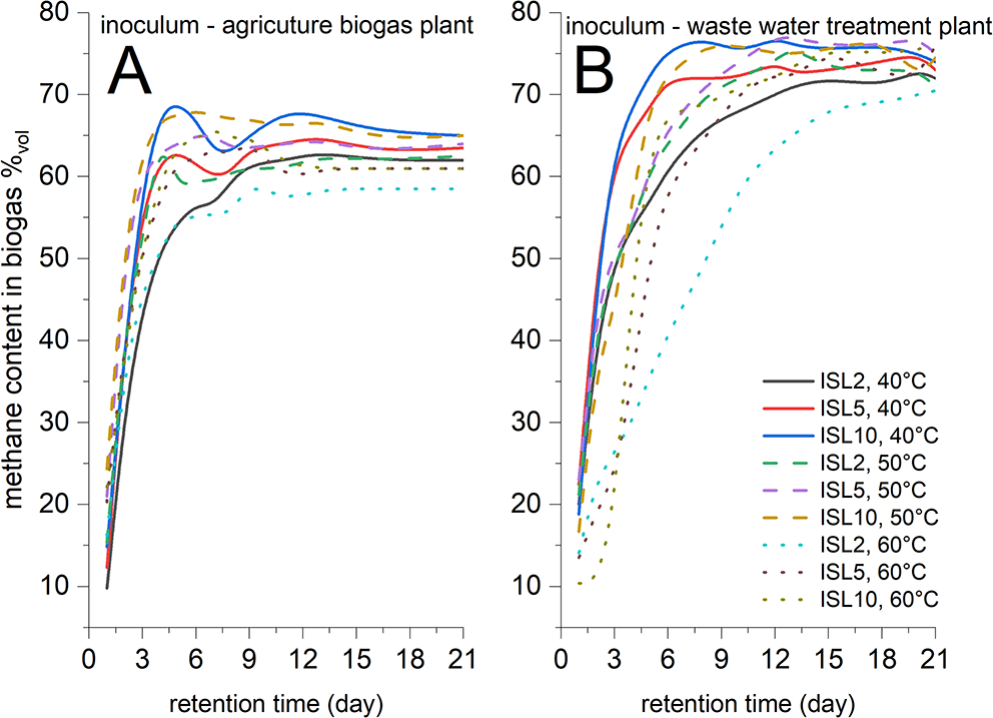
Methane concentration in biogas; (A) inoculum Cejc (agricultural biogas plant); (B) inoculum Modrice (wastewater treatment plant).

The methane content in biogas remained stable after reaching the stationary phase for both inocula. Statistical analysis of methane content in biogas revealed distinct thermal response patterns between inocula (*t* tests, α = 0.05). For the wastewater inoculum (Modrice), only the 50 °C vs 60 °C comparison approached significance (*p* = 0.021), though remaining above the 0.05 threshold, suggesting marginal thermal effects on gas quality. The agricultural inoculum (Cejc) showed no significant temperature-dependent differences in methane content (all *p* ≥ 0.092), indicating stable biogas composition across temperatures. While absolute methane content was consistently higher in the wastewater inoculum (71.0–71.5 vol % vs 61–65 vol % for agricultural inoculum), neither showed statistically significant temperature dependence in biogas composition—a notable contrast to their temperature-sensitive yield patterns. Biogas production and quality generally improved with increasing initial substrate load (ISL), demonstrating that the molasses stillage concentration had no inhibitory effect on fermentation efficiency. The sole exceptions occurred at 60 °C/ISL2, where both inocula exhibited divergent methane concentration kinetics. These results align with literature values Ziemiński and Kowalska-Wentel^27^ 58 vol %; Moraes et al.^28^ 52.1–78.5 vol %, confirming the typical range for stillage-derived biogas. Crucially, while thermal conditions significantly impacted methane yield quantities (particularly for the wastewater inoculum), they exerted minimal influence on the fundamental biogas composition once metabolic stability was achieved.

### 3.1 Microbial Communities

Figure 4 shows the relative abundance of microbial phyla across different temperature conditions (40, 50, and 60 °C) and initial substrate loads (ISL2, ISL5, and ISL10) for two inocula used, Cejc (inoculum from an agricultural biogas plant) and Modrice (inoculum from wastewater treatment plant). Temperatures that are achievable in the field were evaluated. In the case of agricultural biogas plants, we focused on temperatures of 50 and 60 °C, and in the case of technologies for anaerobic stabilization of sewage sludge, on temperatures of 40 and 50 °C.

**Figure 4:**
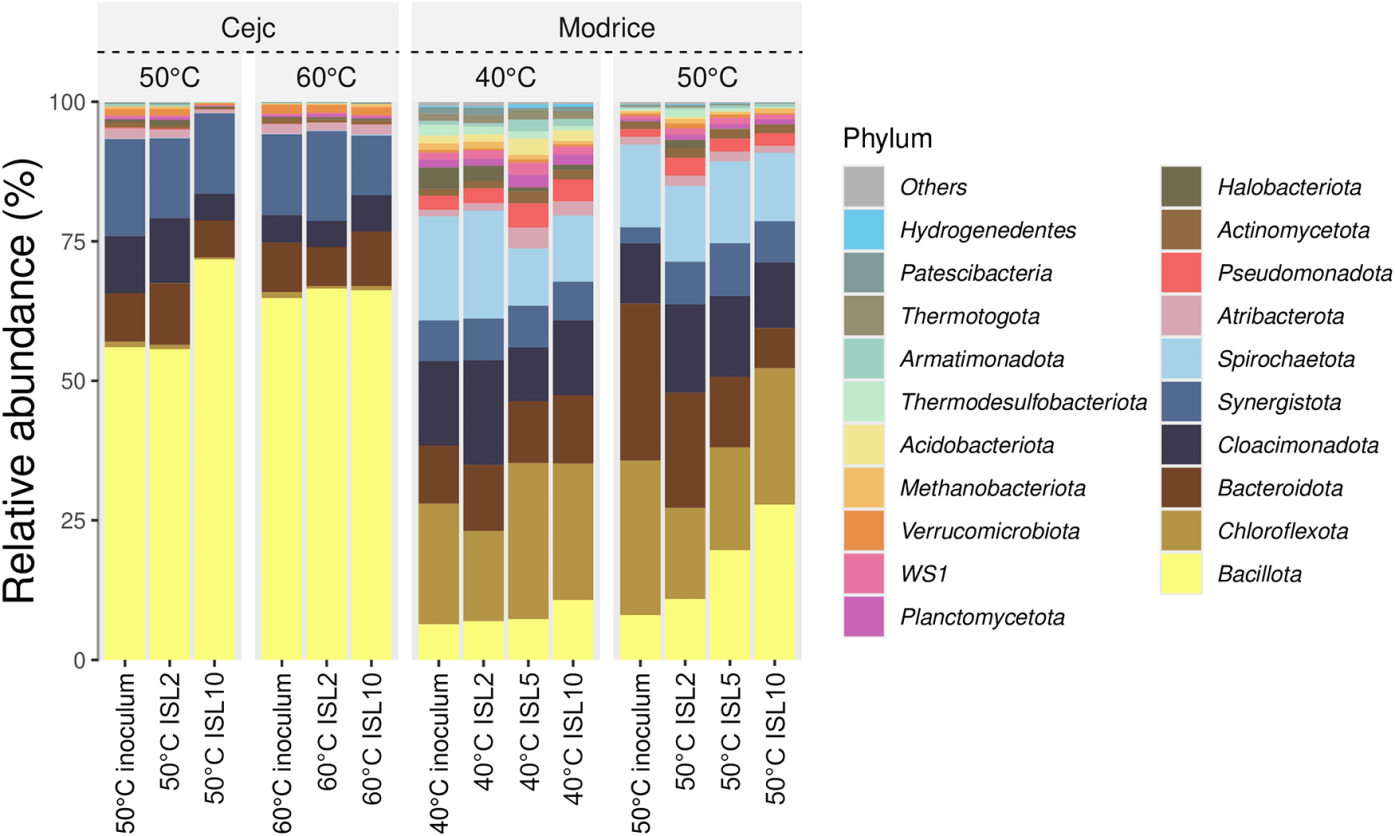
Taxonomic profiles at the phylum level. All phyla shown are at least in one treatment and have more than 0.1% relative abundance. Inoculum Cejc (agricultural biogas plant) and inoculum Modrice (wastewater treatment plant).

The dominant presence of *Bacillota* (yellow) was observed across all samples where the inoculum Cejc (agricultural biogas plant) was used. Significant contributions of *Bacteroidota*, *Cloacimonadota*, and *Synergistota* are also observed, suggesting their active role in fermentation. As temperature and ISL increase, the relative abundance of certain phyla, such as *Bacillota*, *Bacteroidota*, *Cloacimonadota*, and *Synergistota* remains stable, indicating that the community in Cejc is less sensitive to temperature and ISL changes. Overall, the microbial diversity in Cejc appears less diverse (Figure S1), with a few dominant phyla shaping most of the community structure. The dominance of *Bacillota* in inoculum from Cejc across all tested conditions underscores its resilience and adaptability, particularly under thermophilic conditions (50 and 60 °C). This stability aligns with prior research indicating that *Bacillota* includes spore-forming genera capable of withstanding high temperatures and fluctuating organic loading rates.^32^ The relatively stable abundance of *Bacteroidota*, *Cloacimonadota*, and *Synergistota* suggests a less diverse but highly functional microbial community in Cejc. This reduced diversity could reflect the specialized nature of agricultural biogas systems, where a few dominant phyla efficiently perform fermentation and methanogenesis.^33^

Notably, the microbial diversity in the inoculum Modrice (wastewater treatment plant) appears greater than in Cejc, as evidenced by a more even distribution among phyla, such as *Chloroflexota*, *Acidobacteriota*, *Cloacimonadota*, and *Synergistota* alongside *Bacillota*. As seen from the Venn diagram, 170 taxa were shared by both inocula. The shared taxa were comprised of common bacterial genera, such as *Acetomicrobium*, *Clostridium*, *Dethiobacter*, *Syntrophomonas*, and archaeal genera, *Methanobacterium*, *Methanothrix* (Figure S2). This diversity suggests a broader functional repertoire, potentially attributable to the heterogeneous substrates and dynamic conditions typical of wastewater treatment plants.^34^ Greater diversity is often associated with enhanced resilience to environmental changes, as diverse communities can adapt to perturbations by leveraging functional redundancy.^35^ The pronounced response to ISL in Modrice, particularly at higher levels, further emphasizes the flexibility of its community structure compared with the more stable composition in Cejc.

The stability of *Bacillota* and its codominant phyla at higher temperatures and ISLs in Cejc reflects a thermophilic specialization, consistent with studies of biogas reactors operating under high thermal loads.^36^ Conversely, the reduction in diversity observed in Modrice at ISL10 setups, particularly at 50 and 60 °C, suggests that these conditions favor thermophilic and metabolically specialized taxa, such as *Halobacteriota* and *Hydrogenedentes*, while suppressing less competitive phyla. These findings are consistent with research showing that elevated ISLs and longer retention times can impose selective pressures favoring robust, fast-growing species.^37^

Figure 5 depicts the relative abundance of methanogenic genera across different temperatures (40, 50, and 60 °C) and experimental setups (ISL2, ISL5, and ISL10) for two inocula, Cejc (agricultural biogas plant) and Modrice (wastewater treatment plant).

**Figure 5:**
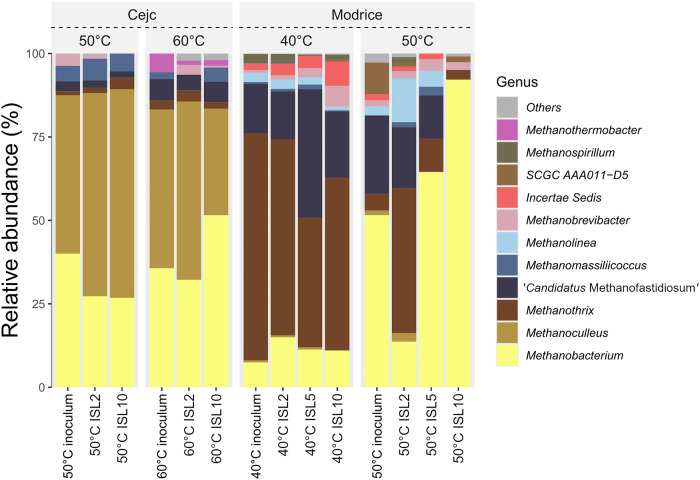
Taxonomic *Archaea* profiles at the genus level. All genera shown are at least in one treatment and have more than 0.1% relative abundance. Inoculum, Cejc (agricultural biogas plant), and Modrice (wastewater treatment plant).

In inoculum from an agricultural biogas plant (Cejc), *Methanobacterium* and *Methanoculleus* genera dominate the methanogenic community, although their relative contributions vary slightly between temperatures and ISL. In inoculum from a wastewater treatment plant (Modrice), *Methanobacterium*, *Methanothrix*, and ‘*Candidatus* Methanofastidiosum’ remain the predominant genera, although their relative contributions vary between temperatures and ISL. However, the overall archaeal diversity in Cejc remains lower than in Modrice, as evidenced by the restricted presence of other genera. The higher baseline diversity in Modrice, particularly at 40 °C, suggests site-specific factors such as nutrient availability, substrate heterogeneity, or inoculum composition, which have been identified as critical determinants of microbial community structure.^38^ Greater diversity may enhance the system’s functional resilience, allowing for more robust adaptation to operational changes.^35^ Conversely, the lower diversity in Cejc reflects a more specialized community, possibly shaped by narrower environmental conditions or a more selective inoculum.

The observed dominance of *Methanobacterium* and *Methanothrix* genera at higher temperatures (50 and 60 °C) corroborates findings from prior studies that emphasize their thermophilic and acetoclastic methanogenic capabilities.^33,39^ The decline in diversity at a higher temperature, particularly in the inoculum Modrice (wastewater treatment plant), aligns with research suggesting that elevated temperatures selectively favor thermophilic genera while limiting less heat-tolerant species. The consistent reduction in diversity under ISL10 setups, irrespective of temperature, indicates that a higher initial substrate load imposes additional selection pressures. This finding agrees with studies reporting that high ISLs favor fast-growing, robust genera while suppressing less competitive or slower-growing species.^37^

Since both inocula were used, the comparison of communities performed by PCoA-plots metric shows obvious clustering driven by axis 1, Figure 6. Inoculum Cejc (agricultural biogas plant) is more compact within the operating temperature. Inoculum Modrice (wastewater treatment plant) shows the formation of two clusters, which are separated according to the operating temperature at 40 and 50 °C (Figure 6).

**Figure 6:**
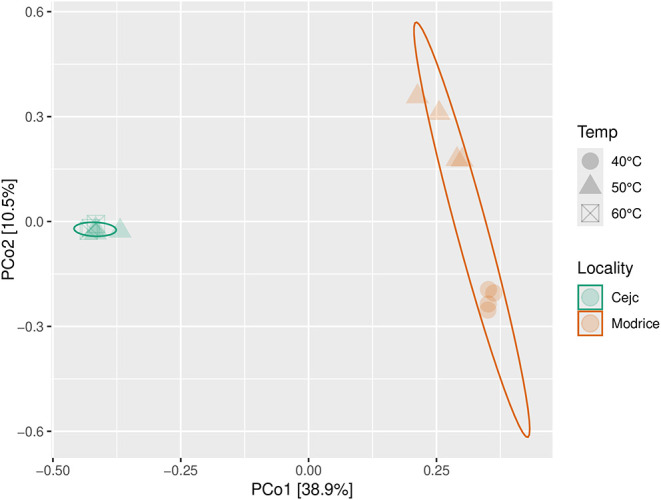
PCoA using Jaccard distance matrix of microbial taxa abundances, data from fermenters operated with different inocula Cejc (agricultural biogas plant) and Modrice (wastewater treatment plant), under different temperatures, and ISL.

### 3.2 Prediction of Functional Pathways

Prediction of the functional microbiological profile of the samples using the FAPROTAX database supports the results that anaerobic and aerobic chemoheterotrophy, methanogenesis, fermentation, and dark hydrogen oxidation are the most abundant metabolic pathways in both samples (Figure 7).

**Figure 7:**
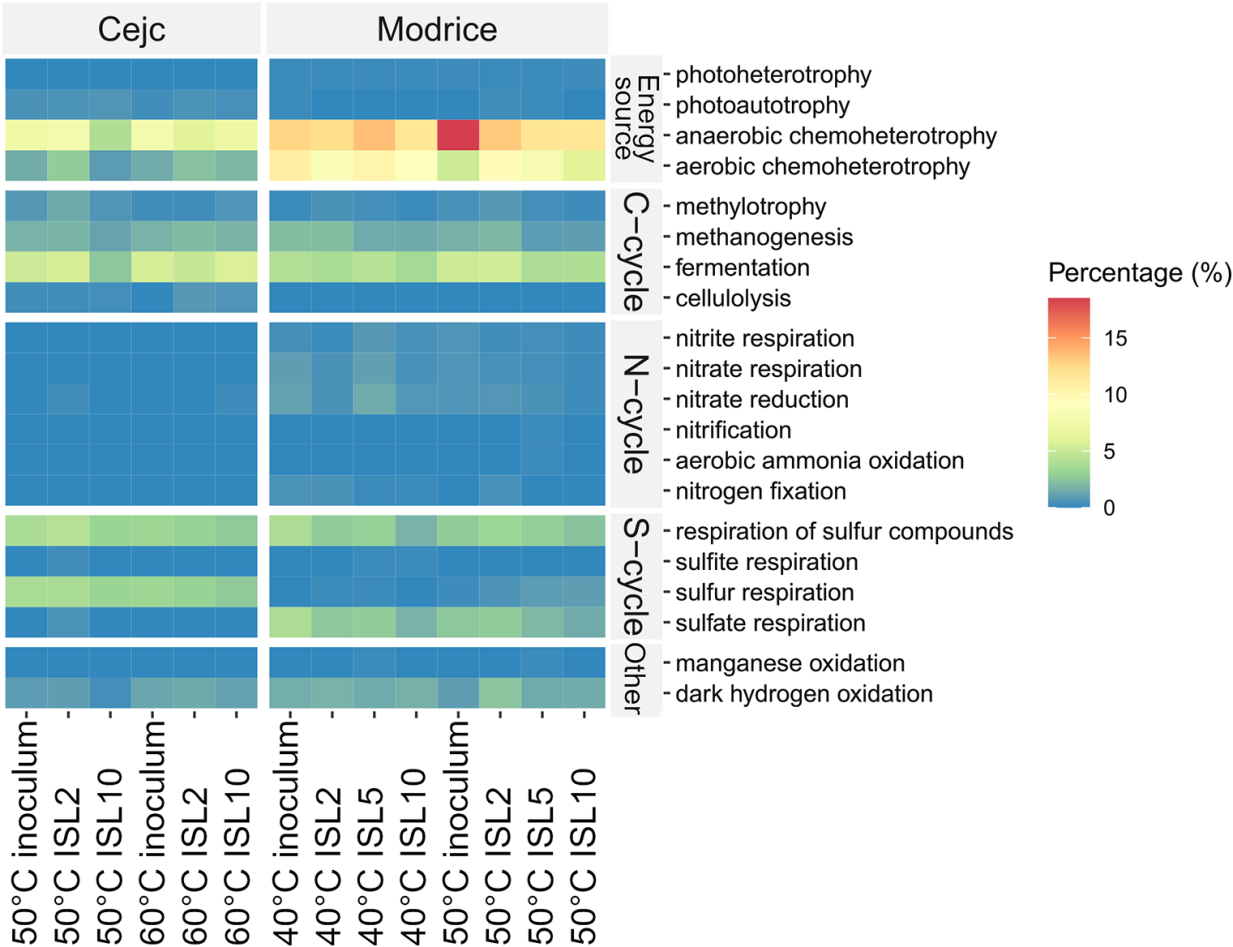
Selected metabolic groups predicted using the database of functional annotations of prokaryotic organisms FAPROTAX.

Our findings align with existing literature that emphasizes the role of temperature and inoculum origin in shaping microbial diversity and metabolic functions. For instance, Angelidaki et al.^33^ demonstrated that higher temperatures favor specialized thermophilic pathways such as methanogenesis, a trend that is evident in the inoculum Cejc (agricultural biogas plant) at 50 and 60 °C, where methanogenic activity dominates. Similarly, the stability of anaerobic chemoheterotrophy across ISLs and temperatures in Cejc is consistent with studies by Liu and Whitman,^39^ which highlighted the robustness of this pathway in agricultural biogas plant communities.

Modrice inoculum (wastewater) showed greater metabolic diversity at 40 °C, supporting enhanced nitrate respiration/reduction vs Cejc, similar findings reported by Narihiro and Sekiguchi,^34^ Demirel and Scherer.^38^ This diversity declined at higher temperatures/ISLs as conditions favored specialized thermophiles Rajagopal et al.,^37^ mirroring typical microbial community shifts under thermal stress.

The heatmap presented in Figure 7 underscores the significant interplay between inoculum origin, operational parameters, and microbial functional potential in anaerobic systems. As demonstrated in recent studies,^40,41^ microbial communities from inoculum Cejc (agricultural biogas plant) and inoculum Modrice (wastewater treatment plant) exhibit distinct metabolic strategies driven by environmental conditions such as temperature and initial substrate load (ISL). Cejc, predominantly a thermophilic system, favors methanogenesis at higher temperatures (50 and 60 °C), consistent with findings from refs 40,42 , which show that thermophilic conditions support the growth of methanogenic archaea and improve methane production efficiency. This is further validated by the dominant presence of methanogenic genera in the Cejc inoculum, suggesting an adaptation to thermophilic environments and enhanced methane generation.

In contrast, Modrice, derived from a mesophilic wastewater treatment plant, exhibits greater metabolic diversity with anaerobic chemoheterotrophy and nitrogen cycling pathways being more pronounced, particularly under mesophilic conditions (40 °C). These findings align with those of Zheng et al.,^43^ who observed increased microbial diversity and metabolic flexibility in wastewater treatment inoculum. The enhanced nitrogen cycling in Modrice, notably nitrate reduction and nitrate respiration, suggests a more complex microbial community capable of utilizing a wider range of electron acceptors. This observation corroborates studies by Zhou et al.,^44^ which highlight the importance of microbial diversity in wastewater treatment systems for effective nitrogen management.

The minimal nitrogen fixation observed across both inoculum suggests that diazotrophic activity does not significantly contribute to system efficiency, a conclusion also supported by recent works such as by Omar et al.,^45^ where limited nitrogen fixation was reported in similar anaerobic setups. Additionally, the weak sulfate respiration and sulfur-related processes detected in both inoculum align with the limited availability of sulfur compounds in anaerobic environments, a finding that echoes research by Pokorná and Zábranská,^46^ which emphasizes the low activity of sulfur-cycling pathways under typical biogas production conditions. The results highlight significant differences in the relative abundance of microbial metabolic functions and ecological traits between the two localities, Cejc and Modrice, suggesting potential environmental or anthropogenic influences shaping microbial community composition (Figure S3).

## 4 Conclusions

The findings of this study emphasize the critical role of optimizing operational parameters, such as the initial substrate load (ISL) and process temperature, to maximize methane yields in anaerobic systems. Notably, the agricultural biogas plant inoculum (Cejc) demonstrated higher resilience to variations in the ISL and temperature, making it more suitable for feedstocks with variable compositions. In contrast, the wastewater treatment plant inoculum (Modrice) required more controlled conditions to avoid yield suppression, particularly at high ISLs and thermophilic temperatures. The study further highlights the suitability of molasses stillage as a substrate for anaerobic digestion, with cofermentation tests revealing superior methane production and negligible hydrogen sulfide concentrations in the produced biogas. When cofermented with sewage sludge, molasses stillage proved to be more effective than typical agricultural feedstocks. The higher methane yields observed in the wastewater inoculum can be attributed to the presence of specific methanogens, such as *Methanothrix* species, commonly associated with anaerobic treatment of wastewater from the distillery industry but less prevalent in agricultural systems.

The comparison between the Cejc and Modrice inocula underscores fundamental differences in the microbial community structure and adaptability. Cejc, dominated by *Bacillota*, demonstrates stability and efficiency under thermophilic conditions, reflecting the importance of core phyla in maintaining system functionality. Conversely, Modrice exhibits greater microbial diversity, which may enhance system stability in response to fluctuating conditions, but is more susceptible to thermal and ISL-related constraints. These findings align with recent advances in microbial ecology, which emphasize the relationship among microbial diversity, community resilience, and operational efficiency in biogas systems.

The study reveals that temperature and ISL are key drivers of methanogenic community composition, with higher temperatures (60 °C) leading to reduced diversity and dominance of thermophilic methanogens, such as hydrogenotrophic *Methanobacterium* and acetoclastic *Methanothrix*. The greater baseline diversity observed in Modrice may reflect site-specific factors, including nutrient availability and initial inoculum composition, conferring resilience to environmental changes. However, this diversity is ultimately constrained by the thermal limits of the microbial community. These findings highlight the dynamic interplay among environmental conditions, operational parameters, and microbial ecology in shaping the functional performance of biogas systems.

Overall, this study reinforces the importance of tailoring biogas system configurations to the specific microbial potential of the inocula and operational conditions. The insights gained contribute to strategies for optimizing biogas production efficiency and stability. Future research should focus on elucidating the mechanistic underpinnings of microbial dynamics and exploring methods to enhance inoculum composition and operational parameter optimization to further improve the sustainability of biogas systems.

## Supplementary Material



## Notes

Comparative α diversity of agricultural and wastewater inocula (Figure S1); Venn diagram (Figure S2); comparative analysis of microbial metabolic functions across localities with emphasis on biogeochemical cycles (Figure S3); and XRF analysis of molasses stillage elements (Table S1) (PDF)
