# Supplementary material for: Biomethane Production from Stillage: The Role of Temperature, Initial Substrate Load, and Inoculum Variability
Source: ACS Omega. 2025 Sep 18;10(38):43687–97. doi: 10.1021/acsomega.5c03798 (PMC12489696; doi:10.1021/acsomega.5c03798)
Supplement: Supplementary file 1 [file ao5c03798_si_001.pdf]

**Biomethane Production from Stillage: The Role of Temperature, Initial Substrate Load, and Inoculum Variability**

Tomáš Vítěz[1,2*], Nikola Hanišáková[2], Pavel Suchý[1], Petra Tejchmanová[1], Jan Kudělka[1], Tomáš Koutný[1], Jan Lochman[3], David Novák[3], Monika Vítězová[2*]

[1]Department of Agricultural, Food and Environmental Engineering, Faculty of AgriSciences, Mendel University in Brno, Zemedelska 1, 613 00 Brno, Czech Republic
[2]Department of Experimental Biology, Section of Microbiology, Faculty of Science, Masaryk University, Kamenice 753/5, 625 00 Brno, Czech Republic; vitezova@sci.muni.cz
[3]Department of Biochemistry, Faculty of Science, Masaryk University, Kamenice 753/5, 625 00 Brno, Czech Republic
* Correspondence: vitezova@sci.muni.cz (M.V.); Tel.: +420-549-497-177 (M.V.)

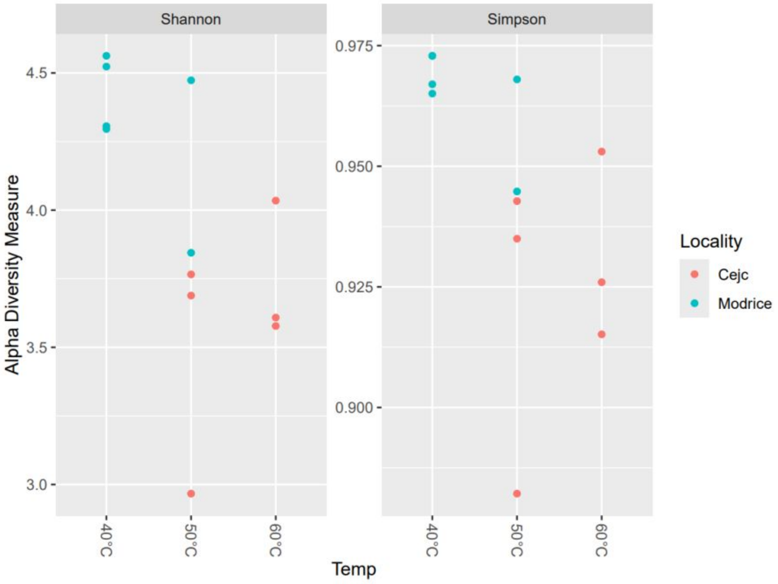


**Figure S1.** Alpha diversity. Inoculum from an agricultural biogas plant (Cejc), inoculum from wastewater treatment plant (Modrice). The graph compares alpha diversity measures (Shannon and Simpson indices) across different temperatures (40°C, 50°C, and 60°C) for two localities, Cejc and Modrice. Shannon diversity values, which account for both species richness and evenness, are consistently higher for Modrice than Cejc, particularly at 40°C, where Modrice shows the greatest diversity (~4.5). As temperature increases to 60°C, a notable decline in Shannon diversity is observed for both localities, indicating a reduction in both microbial richness and evenness at higher thermal stress. The Simpson index, emphasizing species dominance, reflects similar trends. Modrice exhibits higher Simpson values than Cejc, particularly at 40°C and 50°C, implying a more evenly distributed community. However, at 60°C, Simpson values decline for both localities, suggesting that a few taxa become dominant as diversity decreases under thermal pressure. These results highlight Modrice as supporting a more diverse and evenly distributed microbial community compared to Cejc, with temperature exerting a significant selective pressure on microbial composition and structure in both environments.

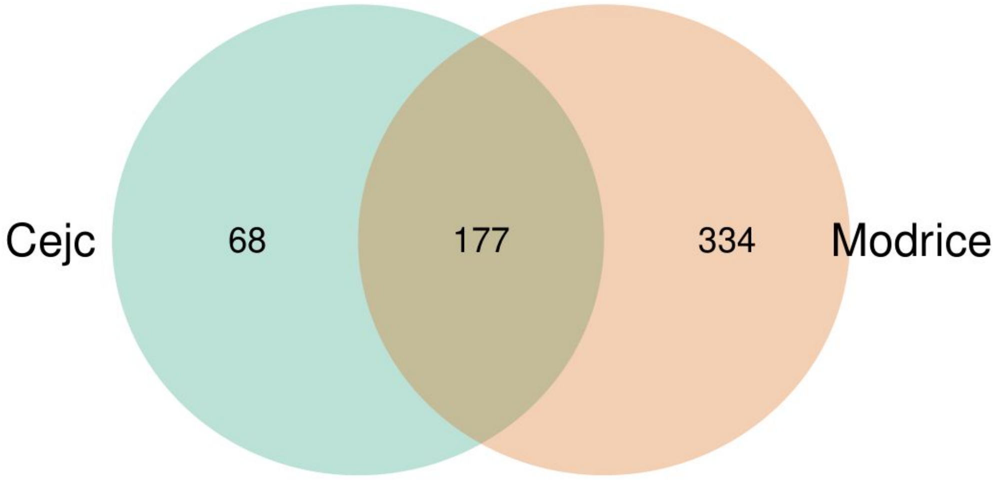


**Figure S2.** Venn diagram of Set 1 of unique taxa belonging to different types of samples portrayed in petal plot. Correlation constructed heatmap of Set 1 different samples type and predicted metabolic pathways predicted by FAPROTAX, using Spearman correlation method (* $p < 0.05$, ** $p < 0.01$, *** $p < 0.001$)

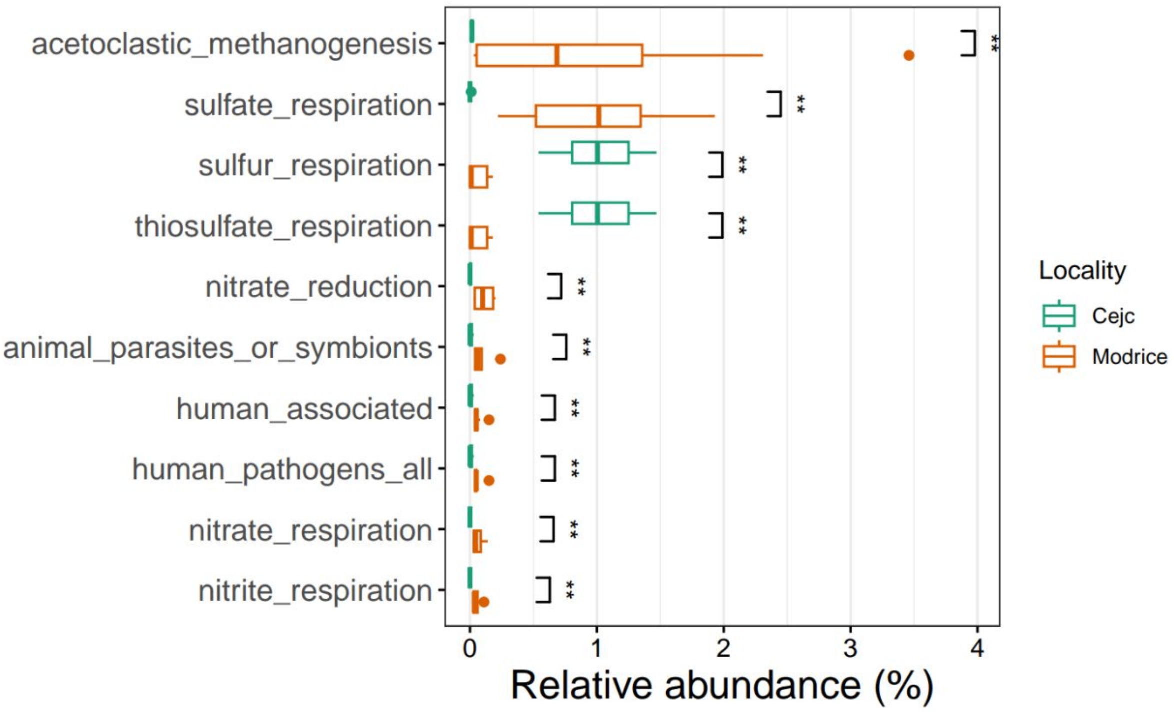


**Figure S3.** Box plot comparing the relative abundance (%) of various microbial metabolic functions or ecological traits between two localities: Cejc (green) and Modrice (orange). Acetoclastic methanogenesis exhibited a significantly higher relative abundance in Modrice, which may reflect localized environmental conditions conducive to methanogenic activity, such as higher organic carbon availability or specific anaerobic conditions. Similarly, functions related to sulfur and nitrogen cycling, including sulfate respiration, sulfur respiration, and nitrate reduction, displayed significant variation between the sites, indicating differing redox conditions or substrate availability. The presence of human-associated and pathogen-related traits, although less abundant, may point to anthropogenic influences or contamination, particularly in Modrice. These findings underscore the importance of environmental context in driving microbial functional potential and warrant further investigation into site-specific factors such as soil chemistry, contamination levels, and land use history.

**Table S1** This table presents the elemental composition of molasses stillage as determined by X-ray fluorescence (XRF) analysis. The XRF technique enables precise screening of elemental constituents, providing essential insights into the chemical makeup of molasses stillage. The results aid in the characterization and understanding of its composition, supporting further research or industrial applications.

| **Element** | **Value [ppm]** | **Element** | **Value [ppm]** |
|---|---|---|---|
| Potassium (K) | 96,454.45±534.67 | Zinc (Zn) | 100.22±9.87 |
| Chlorine (Cl) | 15,192.53±133.63 | Rubidium (Rb) | 67.87±2.12 |
| Sulphur (S) | 12,214.99±198.02 | Copper (Cu) | 36.56±11.55 |
| Calcium (Ca) | 8,253.49±231.19 | Titanium (Ti) | 26.24±8.81 |
| Phosphorus (P) | 1,650.07±183.06 | Antimony (Sb) | 16.16±6.69 |
| Aluminium (Al) | 1,326.41±457.69 | Cadmium (Cd) | 8.89±3.91 |
| Silicon (Si) | 1,061.7±261.66 | Strontium (Sr) | 4.37±1.05 |
| Iron (Fe) | 440.03±50.11 | Palladium (Pd) | 1.93±1.97 |

Mean of measured values (n = 3) is shown; ± Standard Deviation (SD)
